# The Motility Ratio method as a novel approach to qualify semen assessment

**DOI:** 10.1038/s41598-024-79500-1

**Published:** 2024-11-14

**Authors:** Agnès Camus, Charlène Rouillon, Lucie Gavin-Plagne, Eric Schmitt

**Affiliations:** IMV Technologies, ZI no 1 Est, 61270 Saint Ouen sur Iton, France

**Keywords:** Semen motility, Motility Ratio, Accurate analysis, Reliable results, Porcine sperm, Bovine sperm, Animal biotechnology, Animal physiology, Cell biology, Cellular imaging

## Abstract

Many scientific studies often assumed that the most reliable methods for assessing sperm motility are those that give the highest values, and this leads to misinterpretation of the results. This study aims to propose an objective method to validate sperm motility reliability. Bovine and porcine semen samples were split into two equal fractions. Fraction A was kept alive with a motile population considered at maximum proportion, while fraction B was killed with 0% motile population. A range of motile/non motile sperm was performed by mixing both fractions. The Motility Ratio method, comparing measured and theoretical motility, was validated using LEJA slide and IVOS II and applied to other slides. All slides showed strong Concordance Correlation Coefficient between measured and theoretical motility. However, with IVOS II, LEJA slide showed the lowest bias (< 1) while MAKLER or coverslip showed higher bias (> 2 and > 7 respectively) between measured and theoretical motility. This study shows that the best sperm motility is not always the true motility and highlights the importance of implementing a gold standard methodology for motility reliability such as The Motility Ratio method.

**Supplementary Information:**

The online version contains supplementary material available at 10.1038/s41598-024-79500-1.

## Introduction

Numerous studies have tried to evaluate the effects of different treatments or protocols on sperm motility and concentration. However, the results of sperm concentration and motility analyses can be influenced by a large number of factors related to Computer Assisted Semen Analysis (CASA)^1,2^. These factors include the choice and use of analysis slides and supports^2–12^, the settings of the semen analysis devices^13–15^, the type of software used^16^, the analysis time^7^, the number of frames per second during the analysis^7^, the generating trajectories and velocity calculation algorithms^1,17^, the operator variability^18,19^, the sample preparation including semen analysis medium and semen dilution^2,20–25^, or thawing temperature of the straws^26^.

Given the multiple factors that can affect the reliability of sperm analysis results, it is important to standardize the entire semen analysis process with validated methods, from semen sample preparation to analysis.

An important step towards standardization of semen analysis was proposed by O’Meara et al. in 2021^15^. This study aimed to standardize bovine semen analysis setups using CASA to homogenize analyses across laboratories. The reliability of CASA measurements, particularly for motility and concentration, must be verified by gold standards. While gold standards exist for sperm concentration, such as the hemacytometer (World Health Organization WHO, CE Directive EN ISO 23162_2021)^27–29^ or the NucleoCounter^30^, no such gold standard exists for sperm motility analysis. Moraes et al. tried to compare sperm motility and sperm concentration results obtained with different devices, but the method was not sufficiently described and exploited to reach any conclusions^31^. Other studies tried to compare semen analyzers with the manual analysis method, assuming that the manual method is the gold standard^32^. The common point in all these publications is that the reference method used is not justified, and the results of the analyses are often misinterpreted^33–41^. This lack of a gold standard for sperm motility analysis has led to challenges and disagreements within the scientific community. Despite the understanding that sperm motility is crucial for reaching the site of fertilization and penetration of the cumulus and zona pellucida^3,42^, debates about the correlation between motility and fertility remain due to the lack of an objective gold standard for motility measurement^2,42–47^.

In the current situation, it is often assumed that the most reliable methods for assessing sperm quality are those that give the highest values. A decrease in motility is typically interpreted as an adverse effect of a treatment or protocol, with the assumption that motility cannot be improved. For example, the use of capillary-filled slides is not recommended because it gives lower results for total, progressive and velocity motility than the drop and coverslip methods^3,5–9^. This is even described as “clearly underestimated”^4^, although they are alternatively noted to be highly repeatable.

In order to address these challenges and controversies, this paper aims to answer critical questions: How to be sure that the best results reflect reality? What standards can be used to assess sperm motility? How to confirm the accuracy of the measurements? The aim of this study is to answer these questions by developing The Motility Ratio method that follows the standards of validation of analytical methods (International Organization for Standardization—ISO, Food and Drug Administration—FDA, French Agency for Food, Environmental and Occupational Health and Safety—ANSES references) and that can be used in the industries for semen production centers. For this purpose, this method was used with bovine and porcine samples with different slides. The Motility Ratio method consists of preparing a range of samples with known proportion of motile and immotile spermatozoa (theoretical motility). The Motility Ratio method allows to compare the measured and theoretical motility for each kind of microscope slide and CASA systems.

## Materials and methods

All animal procedures conformed to the European regulations: Regulation (EU) 2016/1012^48^ related to zootechnical and genealogical conditions for the breeding, trade in and entry into the Union of purebred breeding animals, hybrid breeding pigs and the germinal products thereof. All semen production centres are approved by the European Directive (EU) 2023/647^49^. Semen samples come from a porcine production centre with approval number F53004P and a bovine production centre with approval number FRCB610. All methods were performed in accordance with these relevant guidelines and regulations. Unless specified otherwise, all consumables and devices were from IMV Technologies (L’Aigle, France).

### Experimental plan

Three motility experiments were conducted with both porcine and bovine samples using CASA.

Experiment 1 was carried out to validate the method of The Motility Ratio. Experiment 2 was a practical example of deviation cases using The Motility Ratio method.

For each experiment, The Motility Ratio method consists of preparing a range of samples with known proportion of motile and immotile spermatozoa. Theoretical and measured motility measurements were compared either using LEJA slide and IVOS II (Experiment 1), slide-coverslip or MAKLER (Sefi Medical instrument, Haifa, Israel) on IVOS II (Experiment 2).

### Semen samples

Sexually mature bulls and boars were collected following procedures^50–52^ of respective semen production centres (Supplementary Table S1).

Bovine samples, from dairy bull breed such as Prim’Holstein and Normand, were fresh or frozen in mini straw (French Cassou straws, 0.23 ml) and were produced for the European market (sorted and conventional straws). Fresh samples were diluted 50 times in OptiXcell or EasyBuffer B using an automated dilutor. Frozen samples were diluted in egg-yolk-based media (sorted semen in home-made medium, and conventional semen in Optidyl) or in OptiXcell (only conventional semen). Semen concentration evaluated on the CASA ranged from 2.7 Million/ml to 142 Million/ml with an average of 30 Million/ml for bovine samples.

Boar samples, from Pietrain and Large White breeds, provided by a French insemination centre, were processed, and analysed up to 48 h after semen collection. Also, only boar semen samples that did not show agglutination were used in this study (Supplementary Figure S2), in order to minimize the bias resulting from this artefact. Samples were either diluted 15 times or up to 35 million per ml in NUTRIXcell Ultra or TRIXcell Plus in 15 ml tubes and stored at + 15 °C. Sperm concentration evaluated on the CASA ranged from 11 to 131 Million/ml with an average of 43 Million/ml.

### Semen sample preparation for The Motility Ratio

Semen samples were split into two equal fractions. Fraction A was kept alive with a motile population considered at the maximum proportion (100% point) while fraction B was killed with a motile population of 0% (0% point).

For bovine sample semen, killing was performed by two rapid successive immersion in liquid nitrogen followed by rapid thawing in a + 37 °C water bath while porcine semen doses were stored in a conventional freezer at -18 °C overnight.

**Fig. 1:**
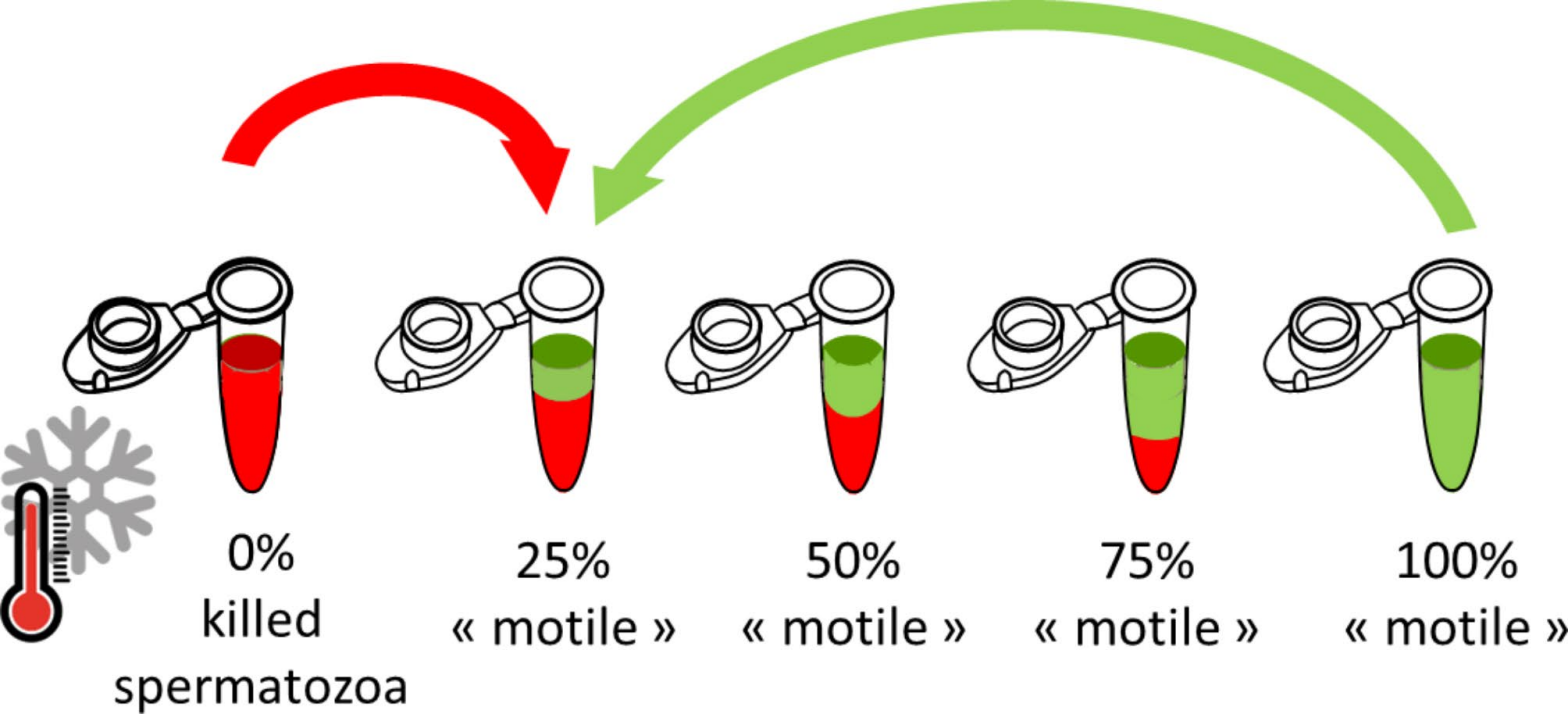
Experiment scheme showing The Motility Ratio method.

A range of 4 or 5 points of Motility ratio was performed at room temperature by mixing Fraction B (immotile) of each sample with either 0%; 20% or 30%; 60% and 100% or 0%; 25%; 50%; 75% and 100% of Fraction A (motile) according to the experiment (Fig. 1, Supplementary Figure S2). Once the range was prepared, samples were kept at room temperature until CASA analysis (up to one hour). The measured 100% point for each sample and each method was used as the reference for calculating the other theoretical points, allowing for each sample to have its own base control. This meant that if a sample had 78% of total motility at its maximum (100% theoretical point), then 25% theoretical point was calculated at 19.5%.

### Semen quality assessment

Semen motility was assessed using IVOS II (Experiment 1 and 2) equipped with a 10× negative phase contrast objective.

Each sample of The Motility Ratio point was aliquoted with 100 µl in 1.5 ml tube. Samples were strictly incubated at + 37 °C for 10 min before analysis. Then, mixed diluted semen was loaded into different slides. For LEJA slide, 3 µl of semen was loaded in a pre-warmed 20 µm chamber depth (Experiment 1 and 3). For Experiment 2, 3 µl of semen was deposited in 10 µm depth chamber MAKLER and 10 µl of semen was deposited on a classical microscope slide where a 22 mm × 22 mm #1 coverslip was placed by dropping it carefully in parallel to the slide over the drop avoiding any air bubbles formation. For each sample, a total of 8 fields were recorded with IVOS II and 2 to 4 replicates were performed. For LEJA slides, those fields were chosen automatically by the system in the predefined area. For LEJA slides and coverslip, they have been chosen in the center of the coverslip. Finally, for MAKLER chamber, the fields were selected outside the grid, as close as possible to the central zone.

The CASA results included a value for total motility (%). Concentration was checked (Million/ml, data not shown). For bovine spermatozoa detection, Qualivet setup was used^15^ while for porcine spermatozoa detection, threshold parameters were as follows: 45 frames were captured at 60 frames/sec, progressive population was defined as VAP > 45 µm/s and STR > 45%, static population was defined as VAP < 15 µm/s and VSL < 0 µm/s.

In order to reduce and avoid analysis biases, all the experiments were performed with two to four independent readings and two experienced lab technicians. All the data were recorded by one technician, with the same lot of slides. The coefficient of variation measured in the lab in 2023 was on average 5% for total motility and concentration on IVOS II and LEJA slides. During motility analyses, lab technicians checked that no drift (static cells moving with the fluid flow) occurred before launching the capture of images.

### Statistical analysis

Statistical analyses were performed on R software (R, version 4.0.2)^53^. Anova test and paired t-test comparison was used to analyze motile sperm velocities in average pathway (VAP) and sperm concentrations between the different points of The Motility Ratio (significant difference if *p* value < 0.05). To assess the accuracy of each method, the bias and the limits of agreement between theoretical and measured total motilities were plotted in a Bland–Altman diagram. Agreement between measured and theoretical motility was plotted in a Concordance diagram. The Concordance Correlation Coefficient (CCC) was shown. Pearson’s correlation was used to calculate the r^2^. Agreement is considered strong and positive when bias is close to 0 and CCC and r^2^ are close to 1 respectively. Total motility (theoretical and measured) for each motility ratio point was also shown in a barplot. Values were expressed as mean ± standard deviation (s.d.).

## Results

### Motile sperm velocities and sperm concentration verification of The Motility Ratio method

Before validating the method, we checked that motile sperm velocities (VAP) were statistically not different between motility ratios above 0% (data not shown), demonstrating that motilities are not affected by The Motility Ratio. Sperm concentration was also verified. With the exception of the slide and coverslip method (p.val = 0.0001), sperm concentrations were statistically identical between motility ratios (data not shown).

### Validation of The Motility Ratio method (Experiment 1)

As a first step, semen total motility, for bovine and porcine samples, was evaluated with LEJA slide and IVOS II to validate The Motility Ratio method.

**Fig. 2:**
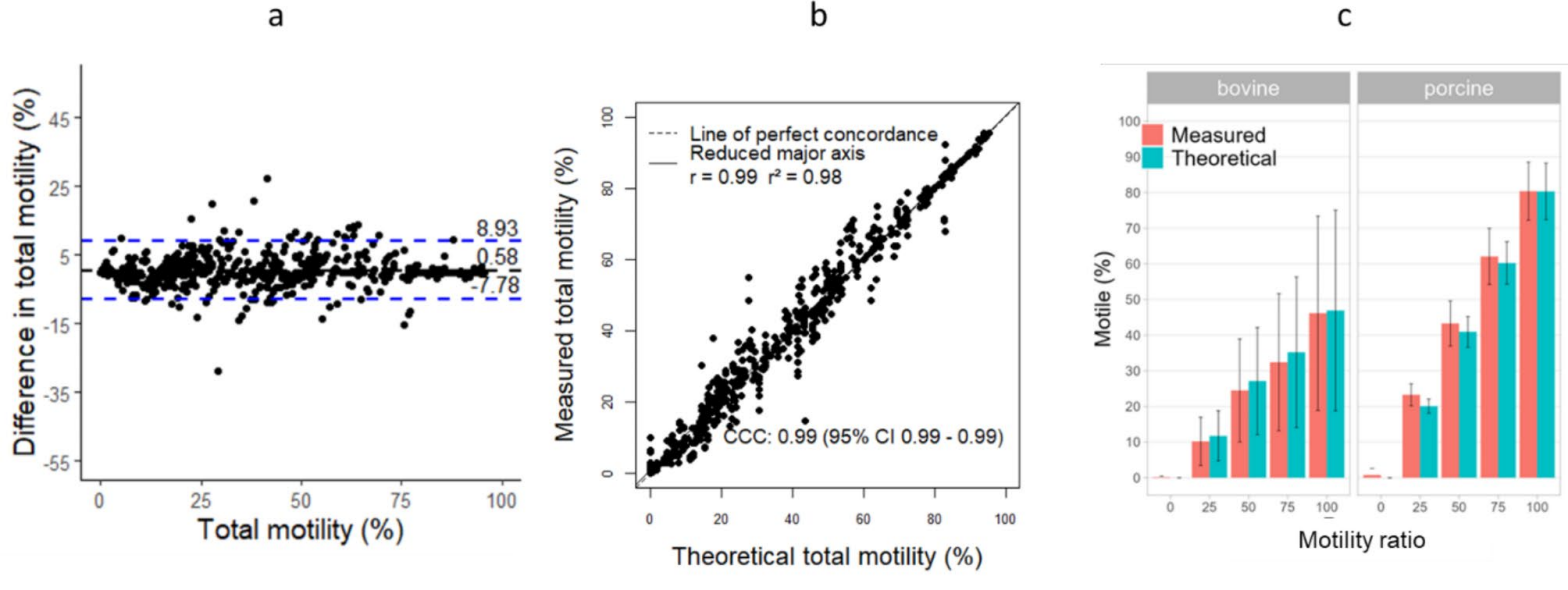
Comparison between measured and theoretical total motility values to assess the accuracy of the method with LEJA slide and IVOS II. Agreement between both motility (Bland–Altman plot) (**a**), concordance and r^2^ correlation (**b**) and motility values of bovine and porcine samples (mean ± standard deviation) (**c**). Dashed black line and dashed blue lines represent the bias and the limits of agreement, respectively (**a**). Dashed black line represents the perfect concordance CCC (**b**). The 5-points motility ratio should show no difference between measured and theoretical motility (**c**).

Calculated bias for total motility with LEJA slide and IVOS II was 0.58, showing low difference between measured and theoretical motility. Concordance and correlation (Pearson) between measured and theoretical motility were considered positive and strong with a CCC = 0.99, r = 0.99 and r^2^ = 0.98 (Fig. 2a,b). These results can be observed in Fig. 2c for the 5-points range 0, 25, 50, 75 and 100% motile sperm cells. The high standard deviation shows the difference of semen quality in samples, particularly in bovine because of the use of frozen-thawed samples (Table 1). When looking at each species, bias were 0.43 and 2.15 for bovine and porcine samples, respectively, and the concordance was still strong for each species with r = 0.99 (Supplementary Table S3 and Figure S4, S5). Indeed, it can be noted that the 25%, 50%, 75% points showed very similar motility values between measured and theoretical (Fig. 2c, Table 1). The 50% point showed measured and theoretical motility of 24.5% and 27.1% in bovine samples, and 43.3% and 40.9% in porcine samples, respectively (Table 1).

**Table 1:** Comparison between measured and theoretical total motility values to assess the accuracy of the method with LEJA slide and IVOS II.

	Motility ratio	Measured		Theoretical	
		Mean (%)	s.d	Mean (%)	s.d
Bovine	0	0.2	0.3	0.0	0.0
	25	10.2	6.7	11.7	7.0
	50	24.5	14.5	27.1	15.0
	75	32.4	19.2	35.2	21.1
	100	46.1	27.3	46.9	28.1
Porcine	0	0.7	1.8	0.0	0.0
	25	23.3	3.1	20.1	2.0
	50	43.3	6.3	40.9	4.3
	75	62.1	7.9	60.2	5.9
	100	80.4	8.1	80.3	7.9

The 5 points motility ratio should show no difference between measured and theoretical motility.

### Application of The Motility Ratio method on different slides (Experiment 2)

Once the method validated, The Motility Ratio method was applied to two other types of slides: slide-coverslip and MAKLER, using an IVOS II.

**Fig. 3:**
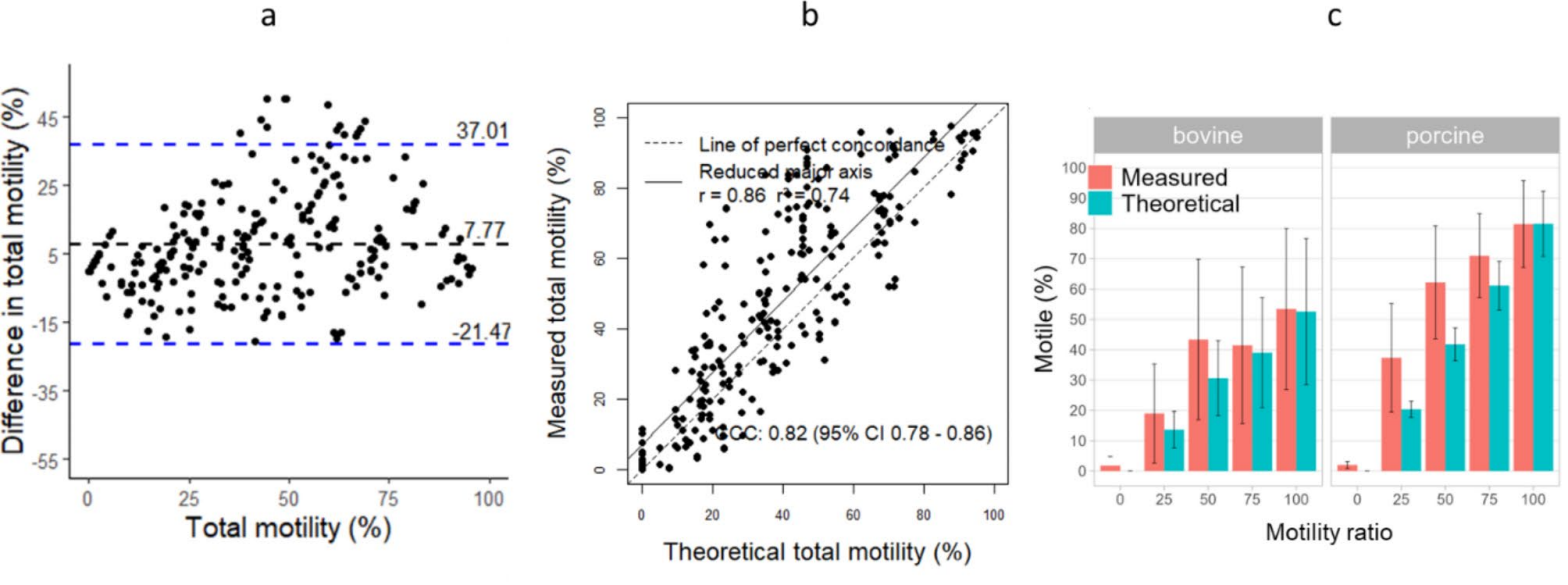
Comparison between measured and theoretical total motility values to assess the accuracy of the slide-coverslip, using IVOS II. Agreement between both motility (Bland–Altman plot) (**a**), concordance and r^2^ correlation (**b**) and motility values of bovine and porcine samples (mean ± standard deviation) (**c**). Dashed black lines and dashed blue lines represent the bias and the limits of agreement, respectively (**a**). Dashed black line represents the perfect concordance CCC (**b**). The 5 points motility ratio should show no difference between measured and theoretical motility values (**c**).

Calculated bias for total motility with slide-coverslip was 7.77, showing intermediate difference between measured and theoretical motility values. Concordance and correlation (Pearson) between measured and theoretical motility were considered positive but intermediate with CCC = 0.82, r = 0.86 and r^2^ = 0.74 (Fig. 3a,b). These results can be observed in Fig. 3 for the 5-points range 0, 25, 50, 75 and 100% motile sperm cells for bovine and porcine samples. The high standard deviation shows the difference of semen quality in samples, particularly in bovine because of the use of frozen-thawed samples (Table 2). When looking at each species, bias were 5.52 and 11.96 for bovine and porcine samples, respectively (Supplementary Table S3 and Figure S6, S7). Concordance was lower in porcine (CCC = 0.72, r = 0.8, r^2^ = 0.64) than in bovine samples (CCC = 0.82, r = 0.86, r^2^ = 0.75). Indeed, slide-coverslip showed over-estimation of the total motility, particularly in porcine samples, with a measured total motility of 62.2% and a theoretical motility of 41.8% for the 50%-point (Fig. 3, Table 2).

**Table 2:** Comparison between measured and theoretical total motility values to assess the accuracy of the slide-coverslip, using IVOS II.

	Motility ratio	Measured		Theoretical	
		Mean (%)	s.d	Mean (%)	s.d
Bovine	0	1.8	3.0	0.0	0.0
	25	19.0	16.3	13.7	6.0
	50	43.4	26.5	30.6	12.4
	75	41.5	25.8	39.0	18.1
	100	53.4	26.5	52.6	24.1
Porcine	0	2.0	1.1	0.0	0.0
	25	37.4	17.9	20.4	2.7
	50	62.2	18.6	41.8	5.4
	75	71.0	13.9	61.2	8.0
	100	81.4	14.3	81.6	10.7

The 5 points motility ratio should show no difference between measured and theoretical motility values.

**Fig. 4:**
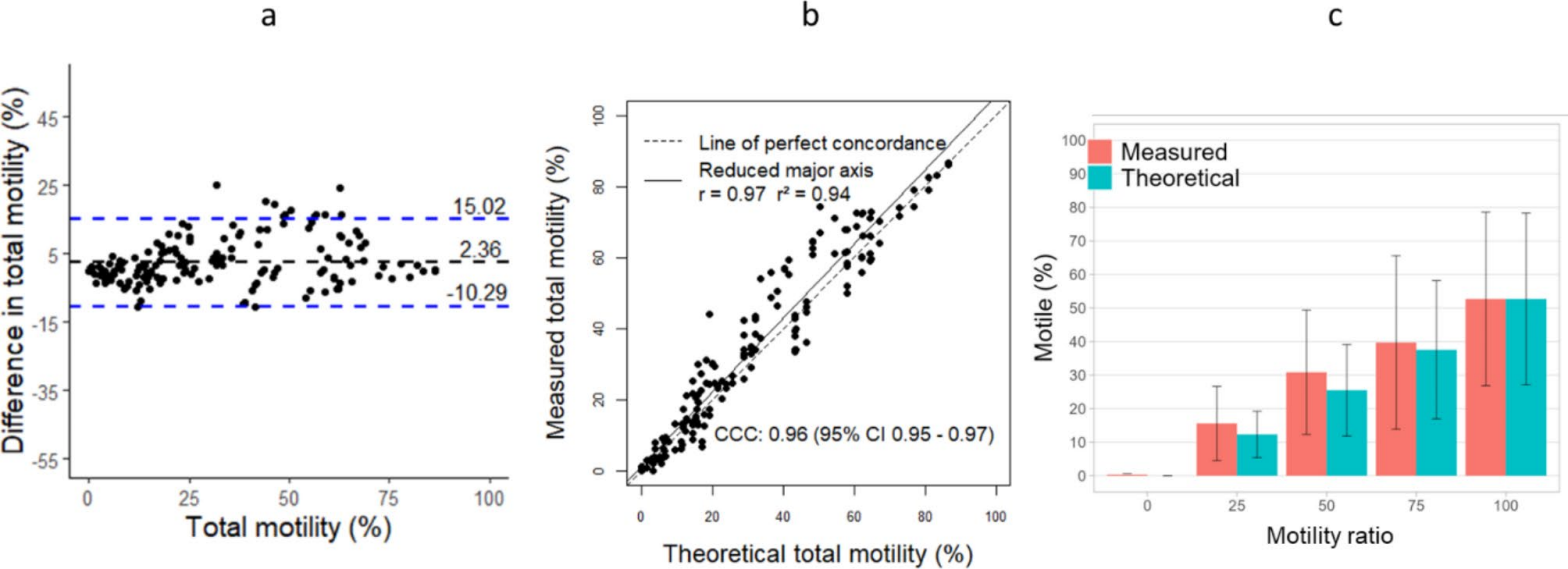
Comparison between measured and theoretical total motility values to assess the accuracy of the MAKLER, using IVOS II. Agreement between both motility (Bland–Altman plot) (**a**), concordance and r^2^ correlation (**b**) and motility values of bovine samples (mean ± standard deviation) (**c**). Dashed black lines and dashed blue lines represent the bias and the limits of agreement, respectively (**a**). Dashed black line represents the perfect concordance CCC (**b**). The 5 points motility ratio should show no difference between measured and theoretical motility values (**c**).

Only bovine samples were used with the MAKLER Chamber. Calculated bias for the motility with MAKLER was 2.36, showing intermediate difference between measured and theoretical motility values. Concordance and correlation (Pearson) between measured and theoretical motility were considered positive and strong with CCC = 0.96, r = 0.97 and r^2^ = 0.94 (Fig. 4a, b). These results can be observed in Fig. 4c for the 5-points range 0, 25, 50, 75 and 100% motile sperm cells for bovine samples. The high standard deviation shows the difference of semen quality in samples, particularly in bovine because of the use of frozen-thawed samples (Table 3). The difference between measured and theoretical motility is most marked at the 50%-point with 30.9% and 25.5% respectively (Table 3).

**Table 3:** Comparison between measured and theoretical total motility values to assess the accuracy of the MAKLER, using IVOS II.

	Motility ratio	Measured		Theoretical	
		Mean (%)	s.d	Mean (%)	s.d
Bovine	0	0.3	0.3	0.0	0.0
	25	15.6	11.0	12.3	6.9
	50	30.9	18.6	25.5	13.7
	75	39.7	25.9	37.6	20.6
	100	52.7	25.9	52.7	25.6

The 5 points motility ratio should show no difference between measured and theoretical motility values.

## Discussion

**Fig. 5:**
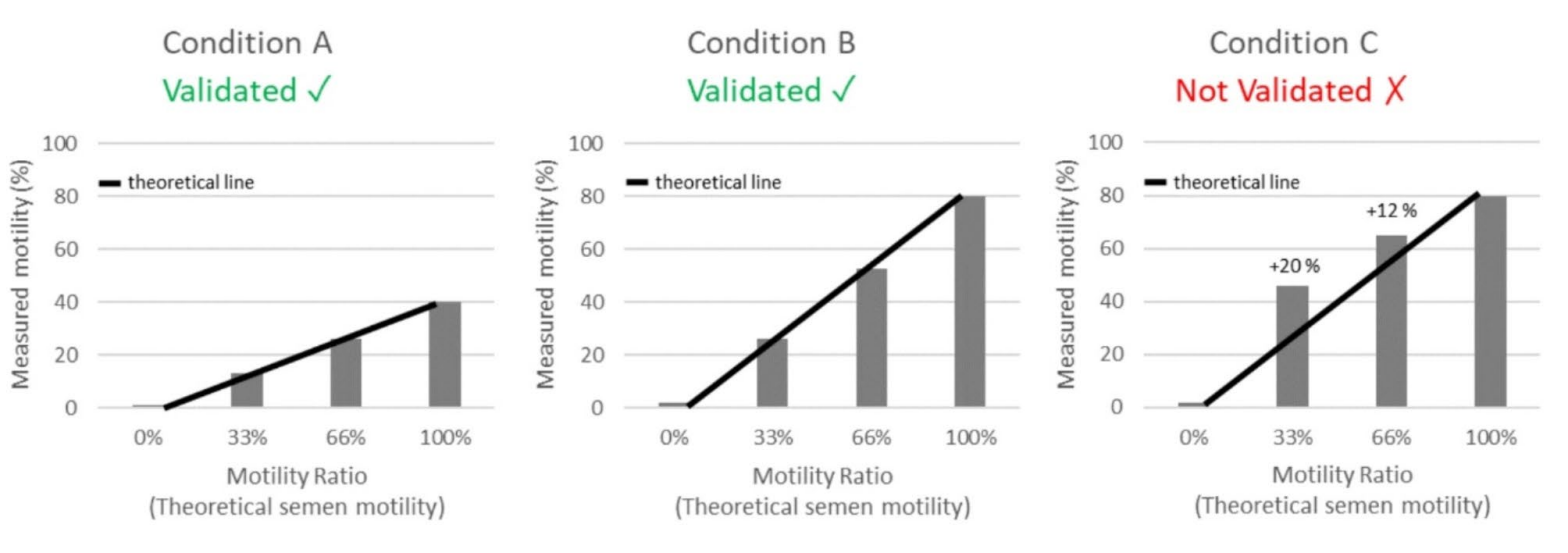
Validation of the method for semen motility assessment.

This study aimed to validate the effectiveness of the developed Motility Ratio method in assessing the reliability of sperm motility results. Our objective was to ensure that the measurements obtained would accurately reflect the theoretical measurements calculated based on the initial quality of the semen samples tested, as illustrated in conditions A and B, in contrast to condition C (Fig. 5). The use of IVOS II with LEJA slides, combined with the protocol used for bovine and porcine semen preparation (storage conditions, The Motility Ratio method and analysis), gave expected results in alignment with the theoretical range without measurement deviation. Thus, we have demonstrated that The Motility Ratio method effectively measures the reliability and accuracy of different semen analysis conditions. However, it is important to note that this method cannot determine the quality of one condition compared to another. For example, condition A may have a lower initial sperm motility than condition B (Fig. 5). Even if both conditions pass the validation of this method, it is not possible to decide which one is more suitable for analysis.

In conditions A and B, the measured motilities follow the theoretical line, thus validating sperm motility measurement methods used in these two conditions. However, if the samples used are the same between the two conditions, as the theoretical 100% point does not have the same starting measured motilities (40% in condition A, and 80% in condition B), it is not possible to decide which method is more appropriate for motility analysis. In condition C, the measured motilities do not follow the theoretical line and are 20% and 12% higher than the theoretical range points 33% and 66% respectively. Also, there is reason to suspect that 100% motility has been over estimated. So, the sperm motility measurement method used in condition C is not validated.

One condition known to affect semen quality is the choice of analysis support. The drop and coverslip methods are often described as better than the capillary chamber method^3–9^. Under the same conditions as method validation, we observed an overestimation of sperm motility compared to the expected theoretical range when using the drop and coverslip analysis support (regular microscope slide and 22 mm × 22 mm coverslip; MAKLER). The degree of deviation increased as the ratio of motile/non motile ratio in the sample increased.

In our experimental design with the drop and coverslip method (regular microscope slide and coverslip; MAKLER), we have shown that the results with highest motility readings are not the most reliable. The overestimation of sperm motility observed with the drop and coverslip method is due to fluid dynamics and rheotaxis rather than measurement drift during the analysis. Our hypothesis is that, once the coverslip is placed on the drop, a flow is created that causes dead spermatozoa to move toward the periphery of the drop due to fluidics. In contrast, motile sperm swim against the flow in response to the flow stimuli and move in the center of the drop, a phenomenon known as rheotaxis^54^. Consequently, a higher quantity of dead sperm in the sample leads to their accumulation at the periphery, concentrating the motile sperm in the center of the preparation.

Other factors can influence the reliability of results, such as the way for filling the slides (filling velocity, type of micropipette tips, among others) or CASA device. To the best of current knowledge, literature is scarce about evaluating the impact of semen filling in slides on motility and sperm distribution. Other CASA can provide reliable sperm motility measurements similar to IVOS II with LEJA slides, with similar or different motility values. The Motility Ratio method could be then used to objectively compare CASA, particularly those with different algorithms for sperm recognition such as artificial intelligence-based CASA^55^ or semen filling methods. Differences could also be attributed to differences in experimental setups, such as the definition of motile, progressive, and static sperm or sample preparation.

## Conclusion

In conclusion, The Motility Ratio method was successfully validated for standardizing sperm motility measurements. It allows the identification of the device, consumables and process used as the missing “gold standard” for sperm motility. This method offers a valuable tool for semen production centers seeking to assess the reliability and accuracy of sperm motility, enhancing devices qualification in semen analysis. Through our study, identified cases of deviation in sperm motility measurements can be attributed to the choice of analysis supports in relation to fluidic and rheotactic phenomena. Thus, depending on the analysis conditions, it has been demonstrated that the percentage of motile sperm cells can be overestimated and therefore the number of potential motile spermatozoa in the dose is overestimated. Furthermore, our study shows that IVOS II in combination with the LEJA slide is a gold standard for sperm motility measurements. It is important to recognize that there are other potential factors that may influence sperm motility. This would enable future research to highlight a potential correlation between sperm quality and fertility.

## Electronic supplementary material

Below is the link to the electronic supplementary material.

Supplementary Material 1

## Data Availability

The raw datasets generated and/or analysed during the current study are not publicly available for intellectual property and confidentiality reasons, but are available from the corresponding author upon reasonable request.
