# Supplementary material for: The Motility Ratio method as a novel approach to qualify semen assessment
Source: Sci Rep. 2024 Nov 14;14:27932. doi: 10.1038/s41598-024-79500-1 (PMC11561241; doi:10.1038/s41598-024-79500-1)
Supplement: Supplementary file 1 — Supplementary Material 1 [file 41598_2024_79500_MOESM1_ESM.docx]

|  | Leja slide  IVOS II | |  | Slide Coverslip  IVOS II | |  | MAKLER  IVOS II | |  |
| --- | --- | --- | --- | --- | --- | --- | --- | --- | --- |
|  | Ejaculates | Observations |  | Ejaculates | Observations |  | Ejaculates | Observations |  |
| EasyBuffer B | 1 | 16 |  | 1 | 16 |  |  |  |  |
| Optidyl | 11 | 182 |  | 7 | 44 |  | 2 | 40 |  |
| OptiXcell | 16 | 222 |  | 2 | 100 |  | 8 | 146 |  |
| NUTRIXcell Ultra | 16 | 194 |  | 10 | 88 |  |  |  |  |
| TRIXcell Plus | 5 | 80 |  |  |  |  |  |  |  |
| Total | 49 | 694 |  | 20 | 248 |  | 10 | 186 |  |

Supplementary Table S1. Number of ejaculates and observations using different analysis support with bull and boar semen


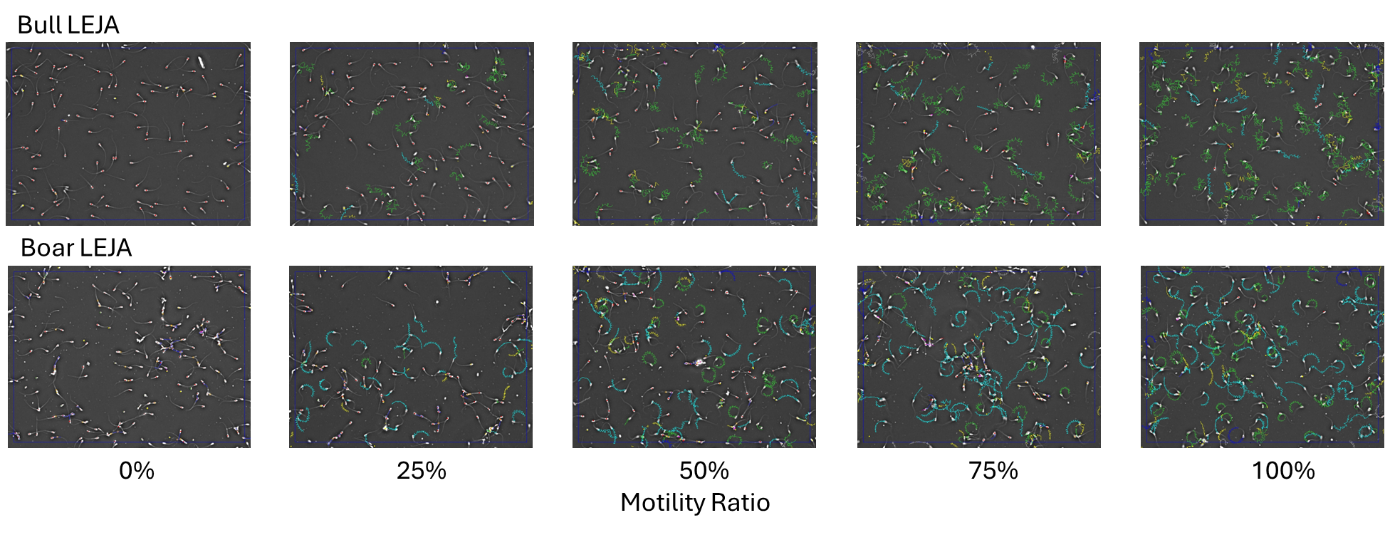
Supplementary Figure S2. Field capture of bull and boar semen sample for each Motility Ratio point in Leja slide

|  | **N Ejaculate** | **N Obs** | **r** | **r²** |  | **CCC** | | |  | **Bland-Altman** | | | | | | | | | | | | | | |  |
| --- | --- | --- | --- | --- | --- | --- | --- | --- | --- | --- | --- | --- | --- | --- | --- | --- | --- | --- | --- | --- | --- | --- | --- | --- | --- |
|  |  |  |  |  |  |  | 95% CI | |  | **Bias** | | |  | **Lower limit of agreement** | | |  | **Upper limit of agreement** | | |  | **t** | **df** | **p** | |
|  |  |  |  |  |  |  |  |  |  | Estimate | 95% CI | |  | Estimate | 95% CI | |  | Estimate | 95% CI | |  |  |  |  |  |
|  |  |  |  |  |  |  |  |  |  |  | Lower | Upper |  |  | Lower | Upper |  |  | Lower | Upper |  |  |  |  |  |
| **Leja slide- IVOS II BULL+BOAR** | 49 | 694 | 0.99 | **0.89** |  | **0.99** | 0.99 | 0.99 |  | **0.58** | 0.26 | 0.89 |  | -7.78 | -8.32 | -7.24 |  | 8.93 | 8.39 | 9.48 |  | 3.6 | 692 | 4.00E-04 | |
| **Leja slide- IVOS II BULL** | 28 | 419 | 0.99 | **0.98** |  | **0.99** | 0.99 | 0.99 |  |  |  |  |  |  |  |  |  |  |  |  |  |  |  |  | |
| **Leja slide- IVOS II BOAR** | 21 | 274 | 0.99 | **0.97** |  | **0.99** | 0.99 | 0.99 |  |  |  |  |  |  |  |  |  |  |  |  |  |  |  |  | |
| **Slide-Coverslip - IVOS II BULL+BOAR** | 21 | 246 | 0.87 | **0.75** |  | **0.82** | 0.78 | 0.86 |  | 7.77 | 5.9 | 9.64 |  | -21.47 | -24.67 | -18.26 |  | 37.01 | 33.8 | 40.21 |  | 8.2 | 245 | 2.00E-14 | |
| **Slide-Coverslip - IVOS II BULL** | 11 | 160 | 0.86 | **0.75** |  | **0.82** | 0.77 | 0.86 |  |  |  |  |  |  |  |  |  |  |  |  |  |  |  |  | |
| **Slide-Coverslip - IVOS II BOAR** | 10 | 86 | 0.8 | **0.64** |  | **0.72** | 0.61 | 0.8 |  |  |  |  |  |  |  |  |  |  |  |  |  |  |  |  | |
| **Makler - IVOS II BULL** | 10 | 186 | 0.97 | 0.94 |  | 0.96 | 0.95 | 0.97 |  | 2.36 | 1.43 | 3.3 |  | -10.29 | -11.89 | -8.69 |  | 15.02 | 13.42 | 16.62 |  | 5 | 185 | 1.00E-06 | |

Supplementary Table S3. Summary of the analysis results of the different experiments using different analysis support, with bull and boar semen.

The numbers of ejaculates (N Ejaculates) and number of observations (N Obs) per experiments is detailed. Person’s correlation coefficient r and coefficient of determination r² between measured and theoretical total motilities values was calculated, as well as the concordance Correlation Coefficient (CCC) and its 95% Confidence Interval (95% CI). Bland-Altman analysis results are detailed with the bias, lower and upper limits of agreement. Agreement is considered strong and positive when bias is closed to 0 and CCC and r² are close to 1.


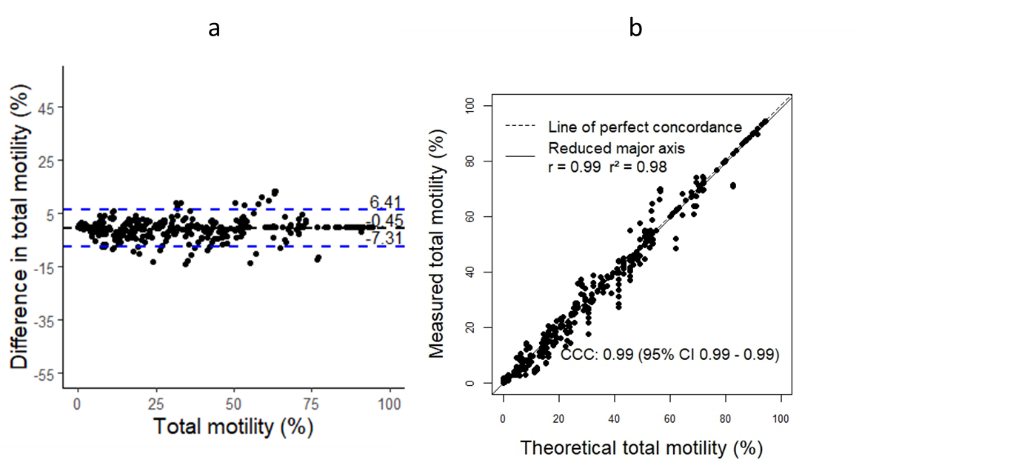


Supplementary Figure S4. Comparison between measured and theoretical total motility values to assess the accuracy of the method with Leja slide and IVOS II.

Agreement between both motility (Bland-Altman plot) (a), concordance and r² correlation (b) and motility values of bovine samples. Dashed black line and dashed blue lines represent the bias and the limits of agreement, respectively (a). Dashed black line represents the perfect concordance CCC (b).


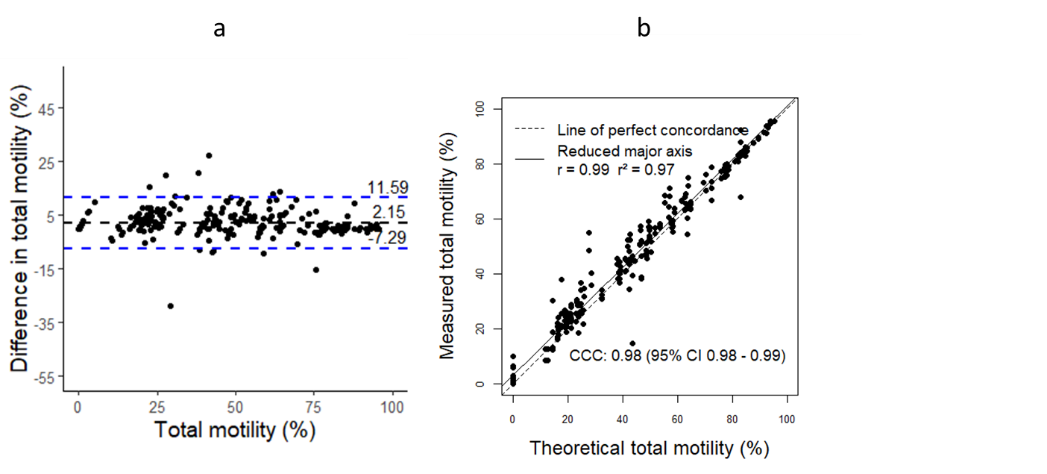


Supplementary Figure S5. Comparison between measured and theoretical total motility values to assess the accuracy of the method with Leja slide and IVOS II.

Agreement between both motility (Bland-Altman plot) (a), concordance and r² correlation (b) and motility values of porcine samples. Dashed black line and dashed blue lines represent the bias and the limits of agreement, respectively (a). Dashed black line represents the perfect concordance CCC (b).


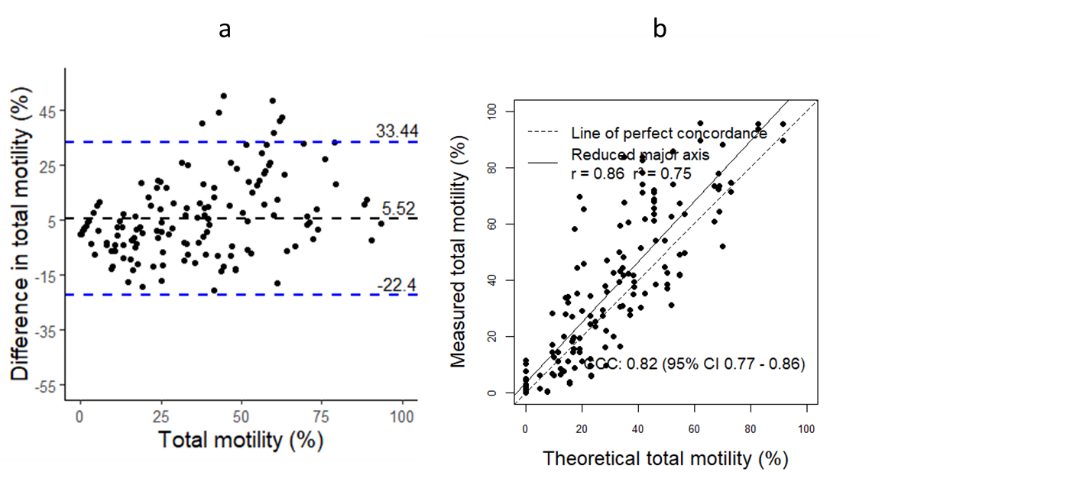


Supplementary Figure S6. Comparison between measured and theoretical total motility values to assess the accuracy of the slide-coverslip, using IVOS II.

Agreement between both motility (Bland-Altman plot) (a), concordance and r² correlation (b) and motility values of bovine samples. Dashed black lines and dashed blue lines represent the bias and the limits of agreement, respectively (a). Dashed black line represents the perfect concordance CCC (b).


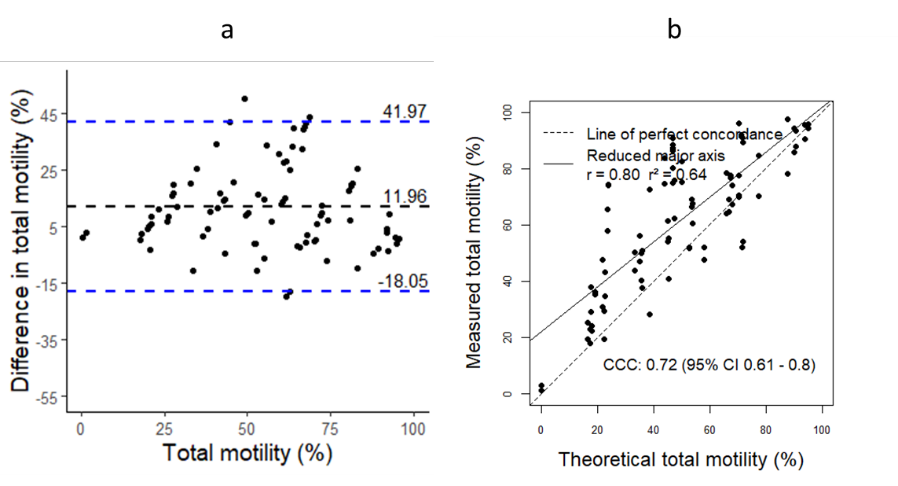


Supplementary Figure S7. Comparison between measured and theoretical total motility values to assess the accuracy of the slide-coverslip, using IVOS II.

Agreement between both motility (Bland-Altman plot) (a), concordance and r² correlation (b) and motility values of porcine samples. Dashed black lines and dashed blue lines represent the bias and the limits of agreement, respectively (a). Dashed black line represents the perfect concordance CCC (b).
